# Supplementary material for: Parametric matrix models
Source: Nat Commun. 2025 Jul 1;16:5929. doi: 10.1038/s41467-025-61362-4 (PMC12220823; doi:10.1038/s41467-025-61362-4)
Supplement: Supplementary file 1 — Supplementary Information [file 41467_2025_61362_MOESM1_ESM.pdf]

# Supplementary Information

| Model | Data Partitioning | Param. | Grid |
|---|---|---|---|
| PMM | fixed random 10% validation | $n$ | 7 |
| | | $r$ | 3 |
| | | learning rate | 0.01 |
| KRR | 10-fold cross-validation | regularization strength | 0.1, 1.0, 10.0 |
| | | kernel function | 'linear', 'rbf', 'poly' |
| | | kernel function parameter | 0.01, 0.1, 1, 10 |
| MLP | | hidden layer sizes | $(50,50)$, $(100)$, $(100,100)$, $(64,32,16)$, $(128,64,32)$, $(8,8,8,8)$, $(16,16,16,16)$, $(32,32,32,32)$, $(8,8,8,8,8)$, $(16,16,16,16,16)$ |
| | | activation function | 'relu' |
| | | regularization strength | 0.0001, 0.001, 0.01 |
| | | learning rate | 0.001, 0.01, 0.1 |
| KNN | | number of neighbors | 3, 5, 7, 11, 17 |
| | | weighting | 'uniform', 'distance' |
| | | leaf size | 10, 15, 20, 25, 30, 35, 40, 45, 50 |
| XGB | | number of estimators | 50, 100, 200, 500, 1000 |
| | | learning rate | 0.01, 0.1, 0.2 |
| | | max depth | 3, 5, 7, 11, 17, 23 |
| SVR | | regularization strength | 0.1, 1, 10 |
| | | kernel function | 'linear', 'rbf', 'poly' |
| | | kernel function parameter | 'scale', 'auto' |
| RFR | | number of estimators | 50, 100, 200, 500, 1000 |
| | | max depth | 'None', 10, 20, 50 |
| | | minimum samples for split | 2, 5, 10, 20, 50 |

**Supplementary Table 1.** Parameters for training on 2D test functions. Independent grid searches were performed for each of the test functions and so each model may have multiple sets of best hyperparameters for this experiment.

| Model | Data Partitioning | Param. | Grid |
|---|---|---|---|
| PMM | fixed random 10% validation | $n$ | 9 |
| | | $r$ | 3 |
| | | learning rate | 0.01 |
| KRR | 5-fold cross-validation | regularization strength | 0.1, 1.0, 10.0 |
| | | kernel function | 'linear', 'rbf', 'poly' |
| | | kernel function parameter | 0.01, 0.1, 1, 10 |
| MLP | | hidden layer sizes | (50,50), (100), (100,100), (64,32,16), (128,64,32) |
| | | activation function | 'relu' |
| | | regularization strength | 0.0001, 0.001, 0.01 |
| | | learning rate | 0.001 |
| KNN | | number of neighbors | 3, 5, 7 |
| | | weighting | 'uniform', 'distance' |
| | | leaf size | 20, 25, 30, 35, 40 |
| XGB | | number of estimators | 50, 100, 200 |
| | | learning rate | 0.01, 0.1, 0.2 |
| | | max depth | 3, 5, 7 |
| SVR | | regularization strength | 0.1, 1, 10 |
| | | kernel function | 'linear', 'rbf', 'poly' |
| | | kernel function parameter | 'scale', 'auto' |
| RFR | | number of estimators | 50, 100, 200 |
| | | max depth | 'None', 10, 20, |
| | | minimum samples for split | 2, 5, 10 |

**Supplementary Table 2.** Parameters for training on polynomial and Fourier series classes of functions. Independent grid searches were performed for each of the two classes of functions and so each model may have multiple sets of best hyperparameters for this experiment.

| Model | Data Partitioning | Param. | Grid |
|---|---|---|---|
| PMM | fixed random 10% validation | $n$ | 7 |
| | | $r$ | 3 |
| | | learning rate | 0.01 |
| KRR | 5-fold cross-validation | regularization strength | 0.1, 1.0, 10.0 |
| | | kernel function | 'linear', 'rbf', 'poly' |
| | | kernel function parameter | 0.01, 0.1, 1, 10 |
| MLP | | hidden layer sizes | (50, ), (50, 50), (100), (100, 100), (64, 32, 16), (128, 64, 32) |
| | | activation function | 'relu','tanh','logistic' |
| | | regularization strength | 0.0001, 0.001, 0.01 |
| | | learning rate | 0.001 |
| KNN | | number of neighbors | 3, 5, 7 |
| | | weighting | 'uniform', 'distance' |
| | | leaf size | 20, 25, 30, 35, 40 |
| XGB | | number of estimators | 50, 100, 200 |
| | | learning rate | 0.01, 0.1, 0.2 |
| | | max depth | 3, 5, 7 |
| SVR | | regularization strength | 0.1, 1, 10 |
| | | kernel function | 'linear', 'rbf', 'poly', 'sigmoid' |
| | | kernel function parameter | 'scale', 'auto' |
| RFR | | number of estimators | 50, 100, 200 |
| | | max depth | 'None', 10, 20, |
| | | minimum samples for split | 2, 5, 10 |

**Supplementary Table 3.** Parameters for training on the NASA Airfoil dataset.

| Model | Data Partitioning | Param. | Grid |
|---|---|---|---|
| PMM | fixed random 10% validation | $n$ | 15 |
| | | $r$ | 3 |
| | | learning rate | 0.01 |
| KRR | 5-fold cross-validation | regularization strength | 0.1, 1.0, 10.0 |
| | | kernel function | 'linear', 'rbf', 'poly' |
| | | kernel function parameter | 0.01, 0.1, 1, 10 |
| MLP | | hidden layer sizes | (50,), (50,50), (100), (100,100), (64,32,16), (128,64,32) |
| | | activation function | 'relu' |
| | | regularization strength | 0.0001, 0.001, 0.01 |
| | | learning rate | 0.001 |
| KNN | | number of neighbors | 3, 5, 7 |
| | | weighting | 'uniform', 'distance' |
| | | leaf size | 20, 25, 30, 35, 40 |
| XGB | | number of estimators | 50, 100, 200 |
| | | learning rate | 0.01, 0.1, 0.2 |
| | | max depth | 3, 5, 7 |
| SVR | | regularization strength | 0.1, 1, 10 |
| | | kernel function | 'linear', 'rbf', 'poly', 'sigmoid' |
| | | kernel function parameter | 'scale', 'auto' |
| RFR | | number of estimators | 50, 100, 200 |
| | | max depth | 'None', 10, 20, |
| | | minimum samples for split | 2, 5, 10 |

**Supplementary Table 4.** Parameters for training on the CERN Dielectron dataset.

| Model | Data Partitioning | Param. | Grid |
|---|---|---|---|
| PMM | fixed 2-point validation | $n$ | 9 |
| | | learning rate | 0.001 |
| MLP | leave-one-out cross-validation | hidden layer sizes | (5,), (10,), (20,), (50,), (5, 5), (10, 10), (20, 20), (50, 50), (10, 5), (20, 10), (100,), (100, 100), (10, 10, 10), (20, 20, 20), (10, 10, 10, 10), |
| | | activation function | 'tanh', 'relu' |
| | | regularization strength | 0.0, 0.0001, 0.001, 0.01 |
| | | optimizer | 'adam', 'sgd' |
| | | learning rate | 0.001, 0.01, 0.1 |
| Polynomial | — | order | 11 |

**Supplementary Table 5.** Parameters for training in the Trotter experiment.

| Model | Data Partitioning | Param. | Grid |
|---|---|---|---|
| PMM | fixed 2-point validation | $n$ | 9 |
| | | regularization strength | 0.01 |
| | | learning rate | 0.001 |
| MLP | 5-fold cross-validation | hidden layer sizes | (100, ), (1000, ), (100, 100), (10, 10, 10), (10, 10, 10, 10), (10, 10, 10, 10, 10), (10, 100, 10), (100, 100, 10), (100, 100, 100), (10, 1000, 10), (100, 1000, 100), |
| | | activation function | 'tanh', 'relu' |
| | | regularization strength | 0.0, 0.0001, 0.001, 0.01, 0.1 |
| | | learning rate | 0.0001, 0.001, 0.01, 0.1, 1.0 |

**Supplementary Table 6.** Parameters for training in the ALMG experiment.

| Name | Equation |
|---|---|
| Franke†[1,2] | $\frac{3}{4}\exp\left\{-\frac{(9x-2)^2+(9y-2)^2}{4}\right\}+\frac{3}{4}\exp\left\{-\frac{(9x+1)^2}{49}-\frac{9y+1}{10}\right\}$ $+\frac{1}{2}\exp\left\{-\frac{(9x-7)^2+(9y-3)^2}{4}\right\}-\frac{1}{5}\exp\left\{-(9x-4)^2-(9y-7)^2\right\}$ |
| Cliff†[1–3] | $\frac{1}{9}\tanh[9(y-x)]+\frac{1}{9}$ |
| Saddle†[1,2] | $\frac{5/4+\cos(27y/5)}{6+6(3x-1)^2}$ |
| Gentle†[1–3] | $\frac{1}{3}\exp\left\{-\alpha\left[\left(x-\frac{1}{2}\right)^2+\left(y-\frac{1}{2}\right)^2\right]\right\}, \quad \alpha=81/16$ |
| Steep†[1–3] | $\frac{1}{3}\exp\left\{-\alpha\left[\left(x-\frac{1}{2}\right)^2+\left(y-\frac{1}{2}\right)^2\right]\right\}, \quad \alpha=81/4$ |
| Sphere†[1–3] | $-\frac{1}{2}+\sqrt{\left(\frac{8}{9}\right)^2-\left(x-\frac{1}{2}\right)^2-\left(y-\frac{1}{2}\right)^2}$ |
| Trigonometric†[2] | $2\cos(10x)\sin(10y)+\sin(10xy)$ |
| Gaussians†[2] | $\exp\left\{-u^2/2\right\}+\frac{3}{4}\exp\left\{-v^2/2\right\}\left[1+\exp\left\{-u^2/2\right\}\right], \quad \begin{cases} u=5-10x \\ v=5-10y \end{cases}$ |
| Cloverleaf†[2] | $\left[\left(\frac{20}{3}\right)^3 uv\right]^2\left[\left(\frac{1}{1+u}\right)\left(\frac{1}{1+v}\right)\right]^5\left[u-\frac{2}{1+u}\right]\left[v-\frac{2}{1+v}\right], \quad \begin{cases} u=\exp\left\{\frac{10-20x}{3}\right\} \\ v=\exp\left\{\frac{10-20y}{3}\right\} \end{cases}$ |
| Cosine Peak†[2] | $\exp\left\{-\frac{2}{5}r\right\}\cos\left(\frac{3}{2}r\right), \quad r=\sqrt{(8x-4)^2+\left(9y-\frac{9}{2}\right)^2}$ |
| Bilinear†[2] | $xy+x$ |
| Vicente Romero†[2,4] | $\frac{6}{5}r+\frac{21}{40}\sin\left(\frac{12\pi}{5\sqrt{2}}r\right)\sin\left[\frac{13}{10}\operatorname{atan2}(y,x)\right], \quad r=\sqrt{x^2+y^2}$ |
| Runge†[2,5] | $\left[(10x-5)^2+(10y-5)^2+1\right]^{-1}$ |
| Fourier series‡ | $\sum_{n=1}^{N}\sum_{m=1}^{M}\left[a_{nm}\sin\left(\frac{n\pi x}{3}\right)\sin\left(\frac{m\pi y}{3}\right)+b_{nm}\cos\left(\frac{n\pi x}{3}\right)\cos\left(\frac{m\pi y}{3}\right)\right], \quad a_{nm},b_{nm}\sim\mathcal{N}(0,1)$ |
| Polynomials‡ | $\sum_{n=0}^{N}\sum_{m=0}^{M}a_{nm}x^n y^m, \quad a_{nm}\sim\mathcal{N}(0,1)$ |

† $(x,y)\in[0,1]\times[0,1]$

‡ $(x,y)\in[-1,1]\times[-1,1]$

**Supplementary Table 7. Collection of mathematical functions used for testing regression performance.** A value, $a$, drawn from a normal distribution with mean $\mu$ and standard deviation $\sigma$ is denoted by $a\sim\mathcal{N}(\mu,\sigma^2)$.

## Supplementary References


1. Franke, R. A critical comparison of some methods for interpolation of scattered data. Tech. Rep. NPS-53-79-003, Naval Postgraduate School, Monterey, California (1979). URL https://hdl.handle.net/10945/35052.
2. Burkardt, J. test_interp_2d. https://people.sc.fsu.edu/~jburkardt/py_src/test_interp_2d/test_interp_2d.html (2016). Accessed: May 2024.

3. McLain, D. H. Drawing contours from arbitrary data points. *Comput. J.* **17**, 318–324 (1974). URL https://doi.org/10.1093/comjnl/17.4.318. https://academic.oup.com/comjnl/article-pdf/17/4/318/1215118/17-4-318.pdf.
4. Romero, V., Burkardt, J., Gunzburger, M. & Peterson, J. Comparison of pure and "Latinized" centroidal Voronoi tessellation against various other statistical sampling methods. *Reliab. Eng. Syst. Saf.* **91**, 1266–1280 (2006).
5. Runge, C. Über empirische Funktionen und die Interpolation zwischen äquidistanten Ordinaten. *Z. Math. Phys.* **46**, 224–243 (1901).
